# Targeted deletion of the aquaglyceroporin AQP9 is protective in a mouse model of Parkinson’s disease

**DOI:** 10.1371/journal.pone.0194896

**Published:** 2018-03-22

**Authors:** Katja Stahl, Soulmaz Rahmani, Agnete Prydz, Nadia Skauli, Nanna MacAulay, Maria N. Mylonakou, Reidun Torp, Øivind Skare, Torill Berg, Trygve B. Leergaard, Ragnhild E. Paulsen, Ole P. Ottersen, Mahmood Amiry-Moghaddam

**Affiliations:** 1 Division of Anatomy, Department of Molecular Medicine, Institute of Basic Medical Sciences, University of Oslo, Oslo, Norway; 2 Department of Neuroscience and Pharmacology, Faculty of Health and Medical Sciences, University of Copenhagen, Copenhagen, Denmark; 3 Centre for Molecular Medicine Norway, Nordic EMBL Partnership, Norway Biotechnology Centre, University of Oslo, Oslo, Norway; 4 Department of Occupational Medicine and Epidemiology, National Institute of Occupational Health, Oslo, Norway; 5 Division of Physiology, Department of Molecular Medicine, Institute of Basic Medical Sciences, University of Oslo, Oslo, Norway; 6 Department of Pharmaceutical Sciences, School of Pharmacy, University of Oslo, Oslo, Norway; 7 Karolinska Institutet, Stockholm, Sweden; Harvard Medical School, UNITED STATES

## Abstract

More than 90% of the cases of Parkinson’s disease have unknown etiology. Gradual loss of dopaminergic neurons of substantia nigra is the main cause of morbidity in this disease. External factors such as environmental toxins are believed to play a role in the cell loss, although the cause of the selective vulnerability of dopaminergic neurons remains unknown. We have previously shown that aquaglyceroporin AQP9 is expressed in dopaminergic neurons and astrocytes of rodent brain. AQP9 is permeable to a broad spectrum of substrates including purines, pyrimidines, and lactate, in addition to water and glycerol. Here we test our hypothesis that AQP9 serves as an influx route for exogenous toxins and, hence, may contribute to the selective vulnerability of nigral dopaminergic (tyrosine hydroxylase-positive) neurons. Using *Xenopus* oocytes injected with *Aqp9* cRNA, we show that AQP9 is permeable to the parkinsonogenic toxin 1-methyl-4-phenylpyridinium (MPP^+^). Stable expression of AQP9 in HEK cells increases their vulnerability to MPP+ and to arsenite—another parkinsonogenic toxin. Conversely, targeted deletion of *Aqp9* in mice protects nigral dopaminergic neurons against MPP^+^ toxicity. A protective effect of *Aqp9* deletion was demonstrated in organotypic slice cultures of mouse midbrain exposed to MPP^+^ *in vitro* and in mice subjected to intrastriatal injections of MPP^+^ *in vivo*. Seven days after intrastriatal MPP^+^ injections, the population of tyrosine hydroxylase-positive cells in substantia nigra is reduced by 48% in *Aqp9* knockout mice compared with 67% in WT littermates. Our results show that AQP9 –selectively expressed in catecholaminergic neurons—is permeable to MPP^+^ and suggest that this aquaglyceroporin contributes to the selective vulnerability of nigral dopaminergic neurons by providing an entry route for parkinsonogenic toxins. To our knowledge this is the first evidence implicating a toxin permeable membrane channel in the pathophysiology of Parkinson’s disease.

## Introduction

Parkinson’s disease is one of the most common neurodegenerative disorders, affecting 1–2% of the population over 50 years [1–4]. This disease is characterized by loss of dopaminergic neurons in the SNpc with decreased dopamine levels in the basal ganglia [5], and is clinically manifested by motor dysfunction, including bradykinesia, resting tremor, rigidity and postural instability, and non-motor symptoms including, but not limited to, cognitive impairment, mood disorders, sleep disorders, constipation, bladder dysfunction and loss of sense of smell [6]. Less than 10% of the cases have a strict familial etiology [7]. For sporadic Parkinson’s disease, the cause of the selective dopaminergic cell death remains unknown, thus restricting the development of drugs which may halt, stop or reverse the degenerative process. However, research has identified a possible link to environmental factors targeting mitochondria, and Parkinson’s disease has been associated with exposure to herbicides, arsenite, and other environmental toxins [8–21]. Mechanisms for the selective vulnerability of dopaminergic neurons to such toxins remain to be elucidated, although there is ample evidence that toxin uptake may occur through the dopamine transporter [22].

Studies have shown that AQP9 like the dopamine transporter DAT, is expressed in TH-positive neurons of the SN in rat, mouse and primates [23–26]. At the subcellular level, AQP9 is localized in the plasma membrane as well as in the inner mitochondrial membrane [23,26]. AQP9 belongs to the aquaglyceroporin subfamily of water channels and is permeable to a broad range of substrates including glycerol and urea [27–29], monocarboxylates (lactate and β-hydroxybutyrate) [28], purines [28], ammonia [30] and arsenite [31].

Here, we show that the parkinsonogenic toxin MPP^+^ permeates *Xenopus* oocytes injected with *Aqp9* cRNA. Second, we demonstrate that stable expression of AQP9 in HEK cells increases their vulnerability to this toxin and to arsenite—another toxin associated with PD. Third, we provide data suggesting that the expression of this water channel contributes to the selective vulnerability of nigral dopaminergic neurons in mice. Specifically, after intrastriatal injections of MPP+, *Aqp9*^-/-^ mice retain a higher number of nigral cells positive for TH than do WT littermate controls. This is the first evidence suggesting that dopaminergic neurons are equipped with a plasma membrane channel that contributes to their vulnerability. Our findings may open new avenues for prevention and therapy.

## Materials and methods

### Heterologous expression in *Xenopus laevis* oocytes and radio-isotope permeability

Oocytes were surgically removed from *Xenopus laevis* frogs (Nasco, USA or National Center for Scientific Research, France) anesthetized with 2 g/l Tricain, 3-aminobenzoic acid ethyl ester,(Sigma-Aldrich A-5040) as previously described [32]. The protocol complies with the European Community guidelines for the use of experimental animals and the experiments were approved by The Danish National Committee for Animal Studies (2015-15-0201-00560).

The cDNA encoding r*Aqp4* and r*Aqp9* (obtained from Søren Nielsen, Aalborg University) were subcloned into the expression vector pXOOM [33]. The constructs were linearized down-stream from the poly-A segments and *in vitro* transcribed to cRNA using mMessage mMachine T7 transcription kit (Ambion, Roskilde, Denmark) followed by transcript purification using MEGAclear (Ambion). Oocytes were micro-injected with 50 ng cRNA with a Nanoject micro injector (Drummond Scientific, Broomall, PA, USA) and kept in Kulori medium at 18°C for 3–6 days prior to experiments.

Five to ten uninjected, AQP4-, or AQP9-expressing oocytes were placed in 0.5 ml uptake solution (in mM: 100 NaCl, 2 KCl, 1 CaCl_2_, 1 MgCl_2_, 10 HEPES (pH 7.4)), and either ^14^[C]urea (1 μ Ci/ml, 9.25 MBq/ml, Perkin Elmer, MA, USA) or ^3^[H]MPP^+^ (3 μ Ci/ml, 1.85 MBq/ml (GE Healthcare, Little Chalfont, England), and incubated with slight agitation at room temperature for 3 minutes. The oocytes were washed in wash solution (in mM: 100 choline chloride, 2 KCl, 1 CaCl_2_, 1 MgCl_2_, 10 HEPES, pH 7.4) to remove residual isotope, and subsequently lysed individually in scintillation vials with 200 μl SDS. Scintillation fluid (Opti-Fluor, Perkin Elmer, Skovlunde, Denmark) was added and the radioactive samples counted (Tri-Carp 2900TR, Perkin Elmer). Data were obtained as counts per minute (CPM)/oocyte and averaged for each construct. As basal uptake displays batch variation, the average CPM/oocyte for each batch of oocytes was normalized to that obtained for AQP4-expressing oocytes and averaged across all seven experiments.

### Cell cultures and MTT cell viability assay

Native HEK cells (ATCC, Manassas, VA) an HEK cells stably transfected with YFP-*h*DAT [34] were cultured in in Dulbecco’s Modified Eagle Medium high glucose (Gibco Life Technologies, Paisley, UK) with 10% fetal bovine serum (Biowest, Nuaillé, France) and 1% Penicillin-streptomycin (Gibco). For HEK cells stably transfected with tetracycline inducible EGFP-*h*AQP9 (pTRE-EGFPAQP9) [35,36], expression of EGFP-*h*AQP9 was induced using 1 μg/ml doxycycline (Clontech Labs, Takara, CA, USA) overnight, and 0.1 mg/ml Hygromycin B (Invitrogen, Carlsbad, CA, USA) and 0.3 mg/ml G418 Geneticin (Gibco) were added for maintenance of the expression.

To confirm protein expression of AQP9 and DAT, WT HEK293 and stably transfected EGFP-*h*AQP9 or YFP-*h*DAT HEK293 cells were plated on coverslips, the cell layer washed with 0.01 M PBS after 24 hours, then fixed in 4% formaldehyde and permeabilized with 0.1% Triton X-100 (Sigma Aldrich, St. Louis, MO, USA) in PBS. Following blocking with10% normal donkey serum in PBS, cells were incubated with the primary antibodies rabbit anti-AQP9 (1:500, AQP91-A, Alpha Diagnostics, San Antonio, TX, USA) or rat anti-DAT (1:800, Merck Millipore, Billerica, MA, USA), and then with donkey anti-rabbit Cy3 or anti-rat Cy2 secondary antibodies (Jackson ImmunoResearch Laboratories, West Grove, USA) diluted 1:1000. Coverslips were mounted with EverBrite ^™^ Hardset Mounting Media with DAPI (4',6-diamidino-2-phenylindole) (Biotium Inc., Fremont, CA, USA). A Zeiss LSM5 PASCAL confocal microscope was used to acquire the images.

HEK293 and YFP-*h*DAT expressing cells were seeded in 96-well plates at a density of 25 000–35 000 per well. HEK293 cells stably transfected with EGFP-*h*AQP9 were seeded in media containing 1μg/mL doxycycline (Clontech Labs, Mountain View, CA, USA) for induction of AQP9 expression at a density of 50 000 cells per well due to impaired growth. The cultures were grown for 24 hours before MPP^+^ iodide (D048, Sigma Aldrich) or arsenite (S7400, Sigma) was added in 100 μL medium at various concentrations.

Cell metabolic activity was assessed by the MTT assay. After 24 hours incubation, 100 μL 0.5 mg/mL MTT reagent was added to each well in the respective media and the plate incubated at 37°C 5% CO_2_ for 3 hours. MTT-containing medium was replaced with 100 μL DMSO (Sigma) and incubated for 30 minutes at room temperature.

Absorbance was measured at 570 nm with a CLARIOstar plate reader (BMG Labtech, Ortenberg, Germany). Blank well values without MTT were subtracted as background.

### Animals

All mice were bred and maintained at the animal facility of the Institute of basic medical sciences, University of Oslo, Norway. *Aqp9*^-/-^ mice were a kind gift from Søren Nielsen’s laboratory, University of Aarhus, Denmark [37]. *Aqp9*^-/-^ mice backcrossed onto a C57BL/6J background (Jacksons Laboratories) and littermate WT mice of mixed gender were used for organotypic cultures (5 days old) and stereotaxic surgery (2–10 months old) All animal experiments were prospectively approved by The Norwegian Animal Research Authority (NARA), project license no FOTS 3730 and 4012, and conducted in accordance with the European Directive 2010/63/EU. Animals were maintained under standard animal facility conditions on a 12 hour light/dark cycle, with food and water available *ad libitum*.

### Organotypic slice cultures of the ventral mesencephalon

WT (n = 8) and *Aqp9*^-/-^ (n = 7) mice were used to prepare organotypic cultures of ventral mesencephalon as previously described [38]. In brief, animals were anesthetized with isoflourane upon decapitation, and the ventral midbrain was isolated and sectioned into slices of 400 μm. The SNpc was unilaterally exposed to either 30 μM MPP^+^ (Sigma Aldrich) diluted in 0.01 M PBS, 60 μM MPP^+^ in PBS followed by 100 μM phloretin (Sigma) diluted in DMSO (Sigma) or only PBS (control) [38,39]. Following fixation in 4% formaldehyde (Sigma Aldrich) overnight, slices were incubated with rabbit anti-TH (1:1000, Chemicon, Billerica, MA, USA) overnight, and then with Cy3 conjugated donkey anti-rabbit (1:1000, Jackson). Images from the SNpc were collected with a LSM 5 Pascal Confocal Microscope (Zeiss, Oberkochen, Germany) using a 20× objective, as previously described [39]. Cells immunostained for TH were counted using ImageJ.

### Intrastriatal injections of MPP^+^

C57BL/6J WT (n = 34) and *Aqp9*^-/-^ mice (n = 29) were deeply anesthetized with zoletil mixture (Zoletil Forte (250 mg/ml), Rompun (20 mg/ml) and Fentanyl (50 μg/ml); 0.1 ml/10 g; intraperitoneally) and then subjected to stereotaxic (TSE systems, Bad Homburg, Germany) intrastriatal injections of MPP^+^ (7.5 μg dissolved in saline) or saline. 1 μl MPP^+^ solution or saline was injected into the striatum 0.6 mm anterior to Bregma, 2.2 mm laterally and 3.2 mm ventrally [40]at 12 μl/hr using a syringe pump (Kd Scientific, Holliston, MA, USA). MPP^+^ was protected against light during the procedure. The injector was left for 5 minutes to allow diffusion before suture. A heating pad (PanLab, Barcelona, Spain) was used to maintain the body temperature at 36.5 ± 0.5°C during the surgery, and the eyes were covered with Vaseline to avoid drying of the cornea.

### Post-operative animal care, behavioral assessments and humane endpoints

The first 24 hrs post-surgery, all animals (total n = 84, MPP^+^: n = 63, saline: n = 21) were put on a heating pad overnight and were treated with Rimadyl (0.1 ml/10 g; subcutaneously every 12 hours) to prevent pain, and a mixture of 0.9% saline and 0.9% sucrose (subcutaneously, every 24 hours) to restore water and energy balance. Animals were fed moisture pellets to improve food- and water uptake. This treatment was continued for as long as needed until the animals were sacrificed 7 days after surgery.

All animals were scored daily to evaluate their post-operative condition. Briefly, animals were assessed daily on a scale from 0–3 for their clinical appearance, including a) weight loss (0%,<5%, 5–10%, 10–15%) b) inactivity (normal, minor changes, less mobile and isolated, inactive) c) external appearance (normal, reduced self-care, nose/eye redness and discolored fur, curved posture and bristling fur) and d) reaction patterns to external stimuli (normal, slightly reduced, moderately reduced and less alert, weak or precomatose) giving a total score ranging from 0–12. Animals reaching a score of ≥10 were euthanized immediately, whereas the remaining animals were sacrificed at post-operative day seven (see following sections). The animal welfare protocol was established in collaboration with the leading veterinary at the animal department of the host institution.

#### Apomorphine rotation test

Six days after the lesion, the animals (n = 70) were treated with apomorphine [41]. The animal was placed in a circular bowl (diameter 20 cm) for 5 minutes to adapt to the environment, then subcutaneously injected with an ice cold and light protected solution of 0.1 mg/kg apomorphine and 0.2 mg/ml ascorbic acid in saline (all from Sigma Aldrich). The animal was video recorded for 15 minutes immediately after the injection. Animals were scored for 360° rotational behavior ipsilaterally and contralaterally to the injection. Data are expressed as net ipsilateral turns (ipsilateral turns—contralateral turns).

### Histological and stereological evaluation of MPP^+^ lesions and TH-positive neurons

Seven days post-surgery, animals (n = 27) were deeply anaesthetized (zoletil mixture; 0.1 ml/10 g; intraperitoneally) and perfusion-fixed with 4% formaldehyde. Brains were dissected, post-fixed, cryo-protected and coronally sectioned into 40 μm. Sections through striatum and midbrain were stained with hematoxylin/eosin for histological analysis (S1 Fig). Successive sections through the SN were immunostained with mouse anti-TH (1:1000, Chemicon) followed by incubation with Streptavidin-Biotinylated horseradish peroxidase complex (1:100; GE Healthcare). Unbiased stereological counting of TH-positive neurons was performed bilaterally in the SNpc, SNpr, and VTA of *Aqp9*^-/-^ and WT mice injected with MPP^+^ (*Aqp9*^-/-^, n = 8; WT, n = 7) or saline (*Aqp9*^-/-^, n = 3; WT, n = 3) as described elsewhere [42,43]. A detailed description of the stereological counting procedure is provided in S1 File.

### High-performance liquid chromatography

HPLC was used to measure the amount of dopamine and its metabolites HVA and DOPAC. WT and *Aqp9*^-/-^ animals (n = 6 for each genotype) were anesthetized with isofluorane and sacrificed by decapitation seven days post-surgery, and the striatum of each hemisphere (MPP^+^ injected and control) were dissected out, flash frozen in liquid N_2_ and stored at -80°C. The tissue samples were homogenized in 250 μl 0.2 M perchloric acid (4°C), mixed with an equal amount of 0.454 μM 2,3-dihydroxybenzoic acid containing 0.135 mM ascorbic acid and centrifuged (20,800 g, 20 minutes, 4°C). The pellet was dissolved in 0.6 ml 0.1M NaOH and protein concentration measured by the BCA Assay (Thermo Scientific, Waltham, MA, USA). The supernatant was mixed with an equal volume of hexane, and allowed 15 minutes to separate (4°C). The bottom layer was collected and filtered through a 0.2 μm nylon filter and stored at -80°C until analyzed for dopamine on a Shimadzu HPLC monoamines analyzer system. The samples were run in duplicates at an isocratic flow rate of 0.8 ml/min.

### RNA isolation and real time qPCR

Seven days post-surgery, animals (MPP^+^, n = 7 for each genotype; saline, n = 3 for each genotype) were decapitated under isofluorane anesthesia. The brains were dissected out and cut along the midsagittal line into the injected and control hemisphere. Midbrain, striatum and neocortex were dissected out from both hemispheres and snap frozen. Total RNA was isolated from the regional brain samples using the RNeasy Mini Kit (QIAGEN, Hilden, Germany) and the relative change in gene expression (ΔΔCt) in the MPP^+^-treated versus control hemispheres of WT and *Aqp9*^*-/-*^ mice, was assessed as described elsewhere [44]. List of the TaqMan probes used in the study are shown in S1 Table and a detailed description of the procedure is available under S1 File.

### Statistics

For stereological, HPLC and qPCR analyses, Student’s *t-test* and Mann–Whitney U tests were performed using SPSS Statistics (Release 24.0.0.2, IBM Corporation, USA). For oocyte experiments, differences between groups were determined with one-way ANOVA and Dunnett’s *post hoc* test in SPSS.

For MTT assays, statistical analysis was performed in SPSS and GraphPad Prism (Ver. 7.03, GraphPad Software, Inc.). OD570 nm was expressed as percentage of the untreated controls. Statistical testing was performed by one-way ANOVA with LSD post hoc tests. Additionally, independent samples *t-tests* were performed comparing each group. Inhibitory concentration 50% values for arsenite were calculated by nonlinear regression, log(inhibitor) vs response (three parameters) and a curve fitted. For log transformed data, the concentration 0 was set to 1 nM.

The behavioral tests were analyzed in R (version 3.2.2). For apomorphine rotation test, ordinary linear regression was used where interaction terms between genotype and treatment were added. To obtain model robust estimates of standard errors,p-values and confidence intervals, we used the *sandwich* function (sandwich package, version 2.3–3) in combination with the *coeftest* function (lmtest package, version 0.9–34). For clinical appearance, a linear mixed model was used, where interaction terms were used to see how the treatment (MPP^+^ vs saline) effect differs between genotypes (*Aqp9*^*-/-*^ vs WT). The mixed model included a random intercept for animals. The variance of random effect was allowed to vary with treatment. The mixed model allowed the variance of the residual term to vary between treatments, and between days.

For the organotypic data, a linear mixed model was applied. The observations come in pairs, one observation for each hemisphere of each slice, where one of the hemispheres is treated (with MPP^+^ or PBS) and the other is untreated. The effect of the treatment is seen as the difference in outcome value between these two sides. An interaction term between treatment, side and genotype was used to estimate how the treatment effect of MPP^+^ and PBS differed between the two genotypes. The outcome values were ln-transformed prior to analysis to obtain a better fit to the normal distribution. Random effects were added for section and slide, with slide nested in section. The residual variance was allowed to vary with treatment and side.

Data were analyzed using the function lme (nlme package, version 3.1–122). A p-value <0.05 was considered statistically significant.

### Experimental design

Data was blinded for analyses susceptible to human bias. For cell quantitation analysis of the organotypic cultures, stereology and behavior tests the investigator was blinded to the groups by third party concealment of treatments.

A minimum of six animals per group was considered as a minimum to estimate statistical power due to within-group variations. The actual number of included animals was calculated based on the high mortality rates following exposure to MPP^+^.

## Results

### AQP9 but not AQP4 is permeable to MPP^+^

**Fig 1:**
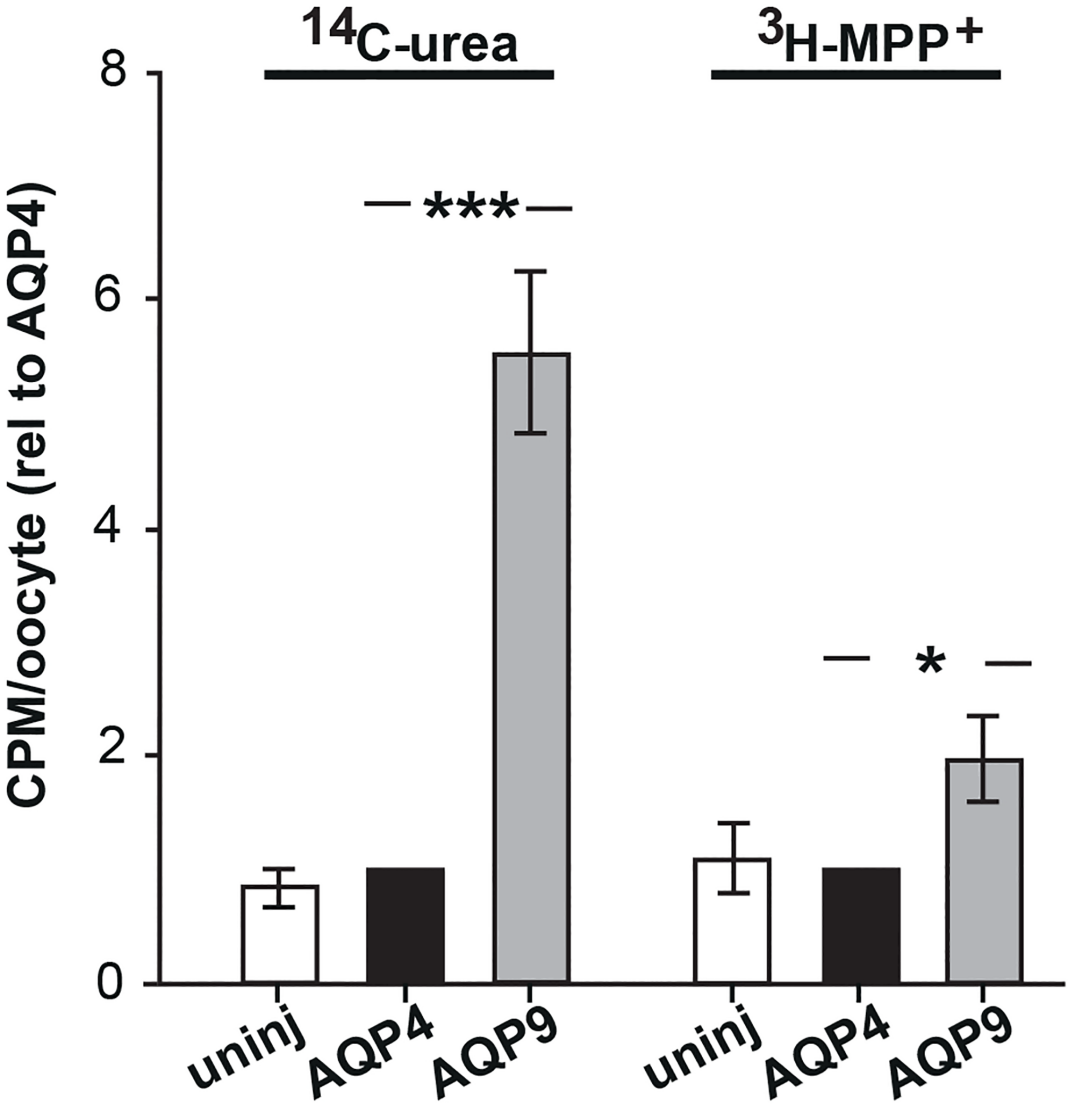
*Xenopus* oocytes expressing AQP9 reveal higher uptake of MPP^+^. *Xenopus* oocytes expressing AQP4 or AQP9 and uninjected oocytes were exposed to ^14^[C]urea or ^3^[H]MPP^+^. Data were obtained as counts per minute (CPM)/oocyte and averaged for each construct. The uptake was normalized to that of the AQP4-expressing oocytes prior to averaging across the n = 7 experiments with five to ten oocytes included for each experimental condition. Compared with uninjected oocytes and AQP4 expressing oocytes, oocytes expressing AQP9 accumulate significantly higher amounts of urea as well as MPP^+^. Bars are mean ± SEM; *p<0.05, ***; p<0.001.

To determine if MPP^+^ is able to permeate AQP9, we expressed AQP9 in *Xenopus* oocytes and exposed these oocytes to radiolabeled MPP^+^ (^3^[H]MPP^+^). As controls, we included uninjected and AQP4-expressing oocytes and determined in parallel uptake of ^14^[C]-urea which is known to permeate AQP9 but not AQP4 [28,45]. Following ^3^[H]MPP^+^ exposure, AQP9-expressing oocytes showed a twofold higher level of radioactivity than AQP4-expressing oocytes (Fig 1). An even larger difference between the two groups of oocytes was found after incubation with ^14^[C]-urea (ratio AQP9 vs AQP4 containing oocytes: 5.51 ± 0.79). Uninjected and AQP4-expressing oocytes did not differ in regard to the level of accumulated ^3^[H]-MPP^+^ or ^14^[C]-urea (Fig 1). In parallel experiments comparing the swelling rates of AQP4-expressing to native oocytes, we confirmed the functionality of the expressed AQP4.

### Stable expression of AQP9 or DAT in HEK cells increases their vulnerability to MPP^+^

**Fig 2:**
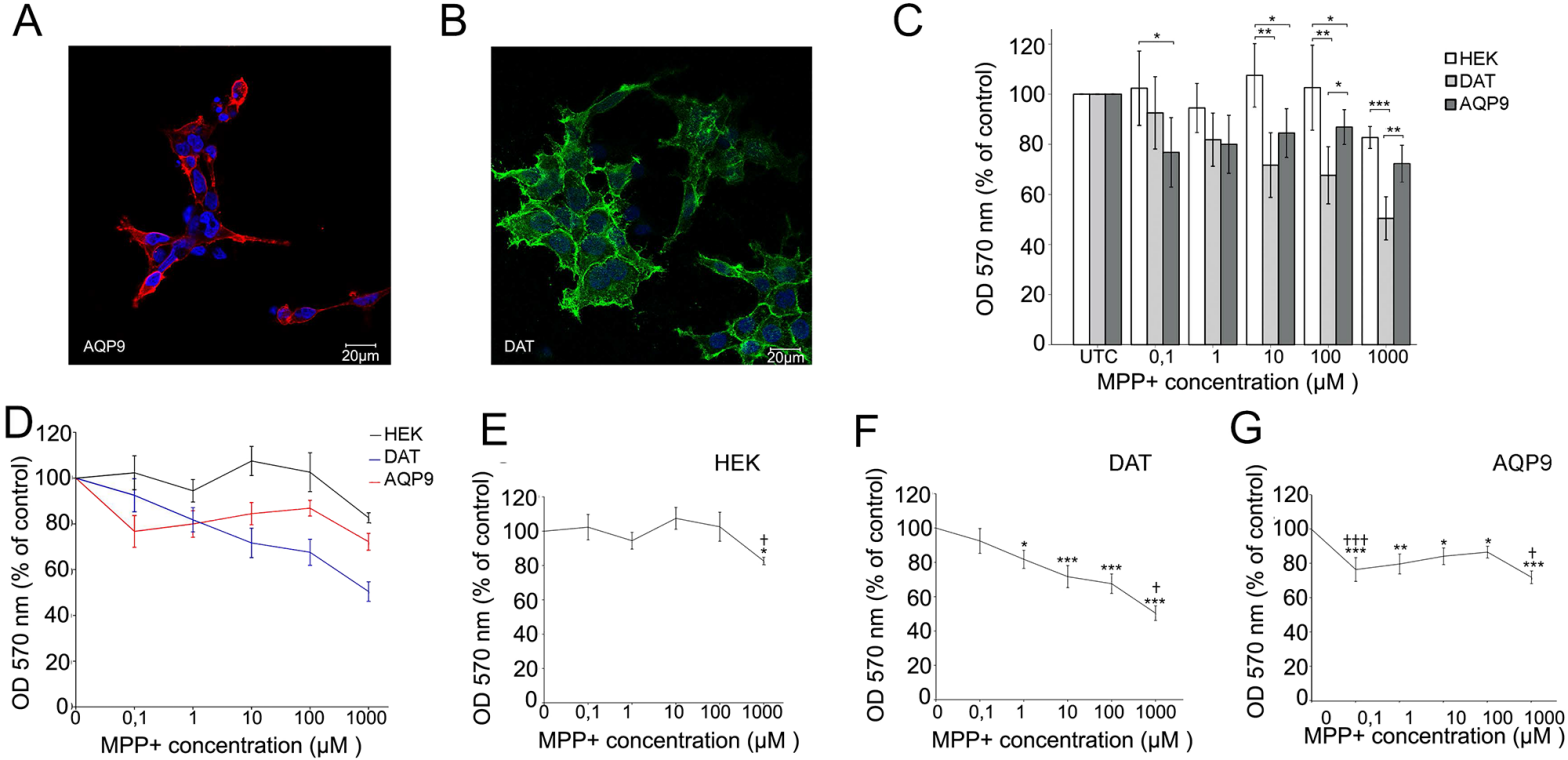
HEK293 cells expressing *h*AQP9 or *h*DAT reveal higher sensitivity to MPP^+^. A-B) Immunofluorescence images of HEK293 cells expressing EGFP-*h*AQP9 (A) or YFP-*h*DAT (B) grown on coverslips confirm plasma membrane localization of the respective constructs (identified by antibodies to AQP9 or DAT). The cells were counterstained with Hoechst to visualize nuclei. C-G) Native HEK293 cells and HEK293 cells expressing EGFP-*h*AQP9 or YFP-*h*DAT were grown in 96-well plates and exposed to different concentrations of MPP^+^ (four wells for each concentration). Cell viability was assessed after 24 hours using the MTT assay. Data were collected from independent plates (n = 3 for each construct) and normalized to respective untreated cells. Native HEK293 cells show sensitivity to MPP^+^ only at very high concentrations (~100 μM). Cells expressing *h*DAT become sensitive at 1 μM MPP^+^, compared with 0.1 μM for cells expressing *h*AQP9. Overlay of the dose/response curve for the three groups (D) and the individual curves for native HEK293 cells (E), YFP-*h*DAT expressing (F) and EGFP-*h*AQP9 expressing HEK293 cells (G) are shown. Bars are mean ± SEM. Asterisks: significantly different from untreated controls; *p<0.05, **p<0.01, ***p<0.001; crosses: significantly different from previous data point: ++<0.01.

**Fig 3:**
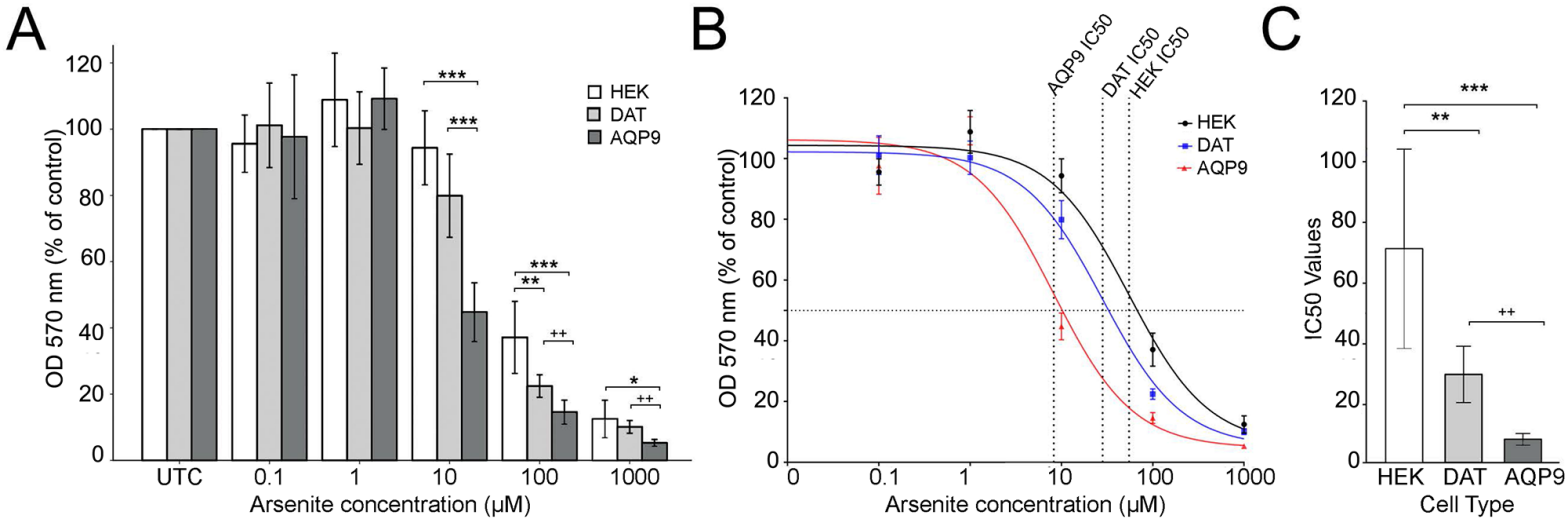
HEK293 cells expressing EGFP-*h*AQP9 are more sensitive to arsenite than HEK293 cells expressing YFP-*h*DAT. A-C) Native HEK293 cells and HEK293 cells expressing EGFP-*h*AQP9 or YFP-*h*DAT were grown in 96-well plates and exposed to different concentrations of arsenite (eight wells for each concentration). Cell viability was assessed after 24 hours using the MTT assay. Data were collected from independent plates (n = 3 for each construct) and normalized to respective untreated cells. Both EGFP-*h*AQP9 and YFP-*h*DAT expressing cells showed higher sensitivity to arsenite, than native HEK293 cells, with EGFP-*h*AQP9 cells being the most sensitive. At the arsenite concentration of 10 μM, stably transfected EGFP-*h*AQP9 were the only cells showing toxin sensitivity (A). The curve showing IC50 values for arsenite calculated by nonlinear regression, log(inhibitor) vs response (three parameters) is shown (B). For log transformed data, the concentration 0 was set to 1 nM. Comparison of the IC50 values shows a significantly lower IC50 value for the HEK293 cells expressing EGFP-*h*AQP9 compared to the native HEK293 cells or HEK293 cells expressing YFP-*h*DAT (C). Bars are mean ± SEM. Asterisks: significantly different from untreated controls; *p<0.05, **p<0.01, ***p<0.001; crosses: significantly different from previous data point: ++ p<0.01.

We compared the toxin sensitivity of HEK293 cells stably expressing YFP-*hDAT* with that of cells stably expressing EGFP-*hAqp9* (Fig 2A and 2B). Neither DAT nor AQP9 is normally expressed in HEK293 cells. Expression of EGFP-*h*AQP9 made these cells sensitive to MPP^+^, as did a stable expression of YFP-*h*DAT (Fig 2C–2G). For *h*AQP9 expressing cells, a statistically significant toxic effect was observed already at 0.1 μM MPP^+^ (Fig 2G). By comparison, an MPP^+^ concentration of 1 μM was required to exert a significant toxic effect on *h*DAT expressing cells (Fig 2F). HEK293 cells expressing *h*AQP9 were clearly more sensitive to the parkinsonogenic compound arsenite than were HEK293 cells expressing *h*DAT (Fig 3).

### *Aqp9* gene deletion protects nigral dopaminergic cells against MPP^+^ neurotoxicity *ex vivo*

**Fig 4:**
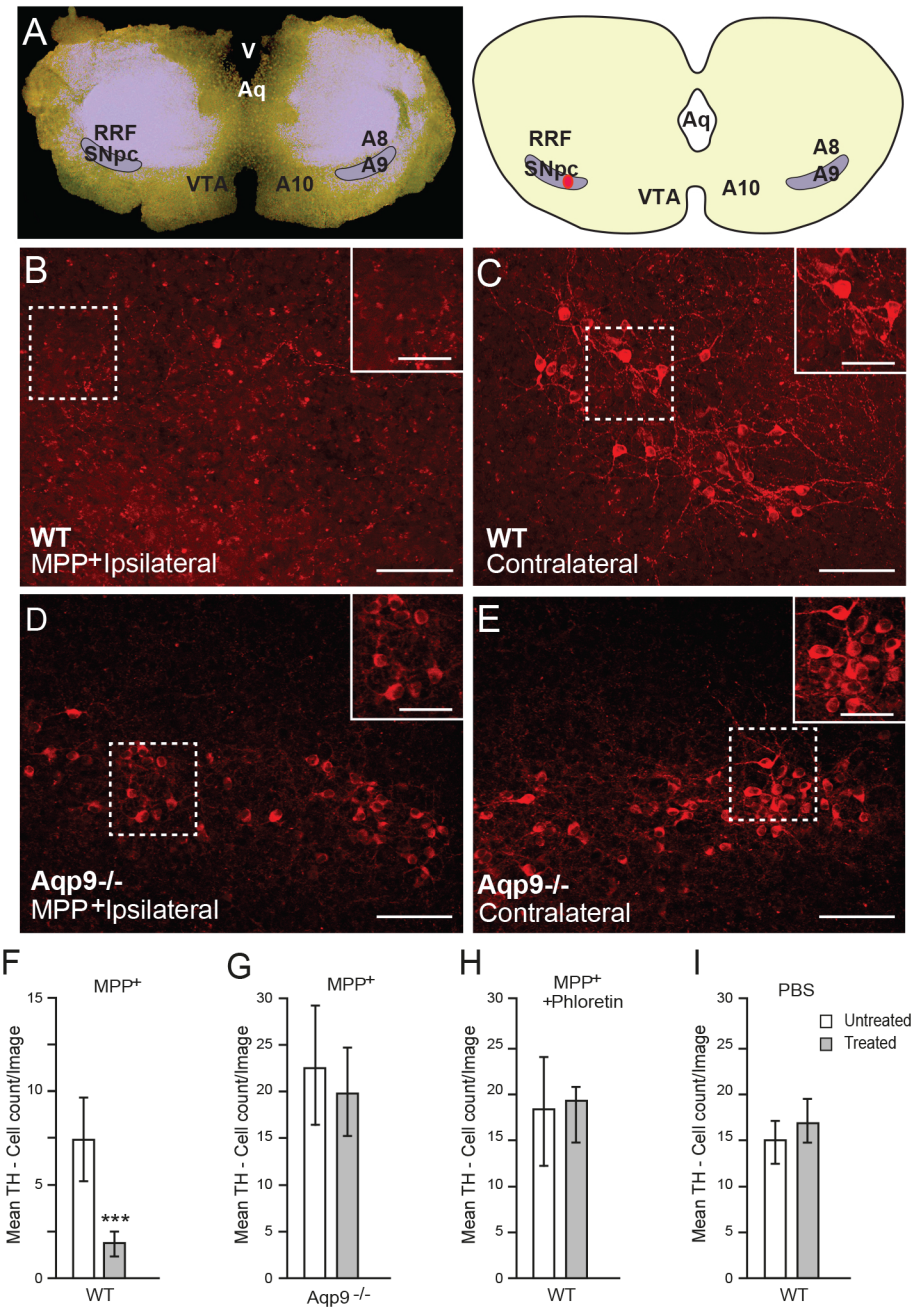
Deletion of *Aqp9* protects against MPP^+^ toxicity *in vitro*. A) Photomicrograph (left) and schematic representation (right) of a representative midbrain slice. Site of MPP^+^ application is indicated in red. B-E) Immunofluorescence staining of representative midbrain slices from WT (B, C) and *Aqp9*^-/-^ mice (D, E) showing TH-positive neurons in the MPP^+^ treated side (ipsilateral; B, D), and in the control side (contralateral, C, E). Note the extensive loss of ipsilateral TH-positive neurons in the WT slice (B). F-H) Quantitative analyses of the TH-positive cell count in slices treated with 30 μM MPP^+^ (F, G) show significant loss of TH-positive neurons in the ipsilateral hemisphere of the WT slice (n = 8) (F). No significant difference was observed between the ipsi- and contralateral hemisphere of *Aqp9*^-/-^ slices treated with 30 μM MPP^+^ (n = 7) (G), WT slices treated with a combination of 60 μM MPP^+^ and 100 μM phloretine (n = 11) (H), or WT slices treated with saline (n = 8) (G). In WT mice, the TH-positive cell count contralateral to MPP^+^ application (F) was lower than the TH-positive cell count in saline treated slices (H), suggesting spillover of MPP^+^ from the ipsilateral side. RRF: retrorubral field; Aq: aqueduct; V: ventricle; A9: population of dopaminergic neurons. Bars are mean ± SEM; ***p<0.001. Scale bar: 100 μm; scale bar inset: 50 μm.

Organotypic slice cultures containing SN were used to assess the effect of MPP^+^ on neurons expressing TH (Fig 4A). To resolve whether knockout of the *Aqp9* gene conferred protection, MPP^+^ was applied unilaterally to midbrain slice cultures from WT and *Aqp9*^-/-^ mice (Fig 4). In slices of WT animals, application of MPP^+^ led to loss of TH-positive neurons on the ipsilateral side (Fig 4B and 4F). Only a few immunopositive elements remained, and most of these were delicate processes that were scattered in the neuropil of SNpc (Fig 4B). In contrast, MPP^+^ did not cause any obvious damage or loss of TH-positive neurons in slices obtained from *Aqp9*^-/-^ mice (Fig 4D). In the latter mice, there was no significant difference in the number of TH-positive neurons between the ipsi- and contralateral sides (Fig 4G). The density of TH-positive neurons contralateral to MPP^+^ exposure was lower in slices of WT mice than in slices of *Aqp9*^-/-^ mice. This might be attributed to spillover of the toxin to the contralateral side.

Application of phloretin—a blocker of AQP9—mimicked the effect of *Aqp9* gene deletion. Thus, in the presence of 100 μM phloretin, MPP^+^ did not cause any significant loss of TH-positive neurons (Fig 4H). Application of PBS in lieu of MPP^+^ did not cause any cell loss (Fig 4I). In sum, these experiments indicated that deletion of *Aqp9* protects against MPP^+^ toxicity *in vitro*.

### *Aqp9* gene deletion protects nigral dopaminergic cells against MPP^+^ neurotoxicity *in vivo*

#### Stereological cell counts in the ventral midbrain

**Fig 5:**
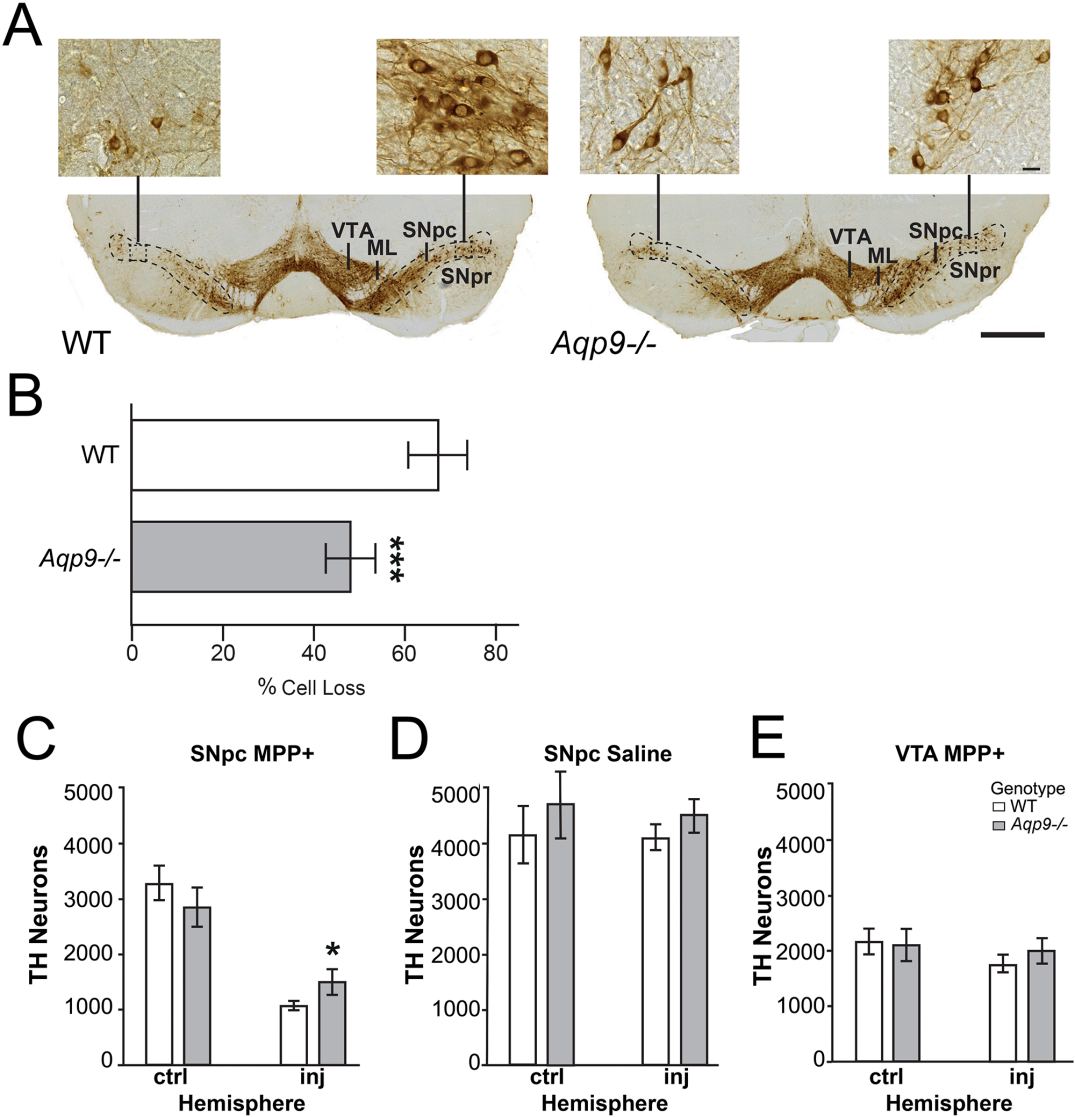
Deletion of *Aqp9* protects against MPP^+^ toxicity *in vivo*. A) Representative midbrain sections of WT and *Aqp9*^-/-^ mice, seven days after treatment with unilateral striatal injection of 7.5 μg MPP^+^. Cell bodies and processes of the dopaminergic neurons are identified by TH immunostaining. The visible reduction of the TH immunostained cell bodies in the ipsilateral SNpc is more pronounced in WT than in *Aqp9*^-/-^ mice. B) Quantitation of the TH-positive neurons in SNpc is described in the text. TH-positive cell loss, calculated as [(n contra—n ipsi): (n contra)] is significantly lower in *Aqp9*^-/-^ mice, where 47.59% of the cells are lost, compared to 67.02% in WT littermates (p<0.001). C) Compared with WT mice, *Aqp9*^-/-^ mice show a significantly higher count of TH-positive neurons on the injected side (p<0.001). D) Animals treated with saline showed no significant loss of TH-positive cells in SNpc, regardless of genotype. E) In WT mice, VTA showed a slight but statistically significant decrease in number of TH-positive neurons on the ipsilateral side (p = 0.033). No change was observed in VTA of *Aqp9*^-/-^ mice. The MPP^+^ treated group consisted of 15 animals (*Aqp9*^-/-^, n = 8; WT, n = 7), and the saline group consisted of six animals (*Aqp9*^-/-^, n = 3; WT, n = 3). ML, medial lemniscus. Bars are mean ± 2 SEM; n = 6; *p<0.05, **p<0.01, ***p<0.001. Scale bar, 1000 μm; scale bar inset, 20 μm.

*Aqp9*^-/-^ and littermate WT mice were subjected to unilateral intrastriatal injections of MPP^+^ or saline (control). The density of dopaminergic neurons was quantified in the SNpc, SNpr and VTA of *Aqp9*^-/-^ (n = 8 for MPP^+^, n = 3 saline) and WT animals (n = 7 for MPP^+^, n = 3 saline) by unbiased stereological counting of TH-positive cells in each region [46]. Cell counts for SNpc and VTA are shown in the S2 Table. Gundersen coefficients of error m = 1; [43] ranged from 5–9% for both SNpc and VTA. Both genotypes showed a significant reduction in the number of TH-positive neurons in the ipsilateral SNpc following MPP^+^ injections (S2 Table, Fig 5A–5C). In the ipsilateral SNpc, the reduction in TH cell number (using the cell count in the contralateral hemisphere as reference) was significantly lower in *Aqp9*^-/-^ animals than in WT littermates (47.59% and 67.02%, respectively; p<0.001) (Fig 5B). In the ipsilateral VTA we found a small but significant reduction in TH-positive neurons in WT animals, but no reduction in *Aqp9*^-/-^ mice (Fig 5E). As expected, SNpr contained few TH-positive cells in either mouse line. No significant change was observed in animals injected with saline, regardless of genotype (p>0.05) (Fig 5D).

#### Clinical appearance and behavioral assessment

**Fig 6:**
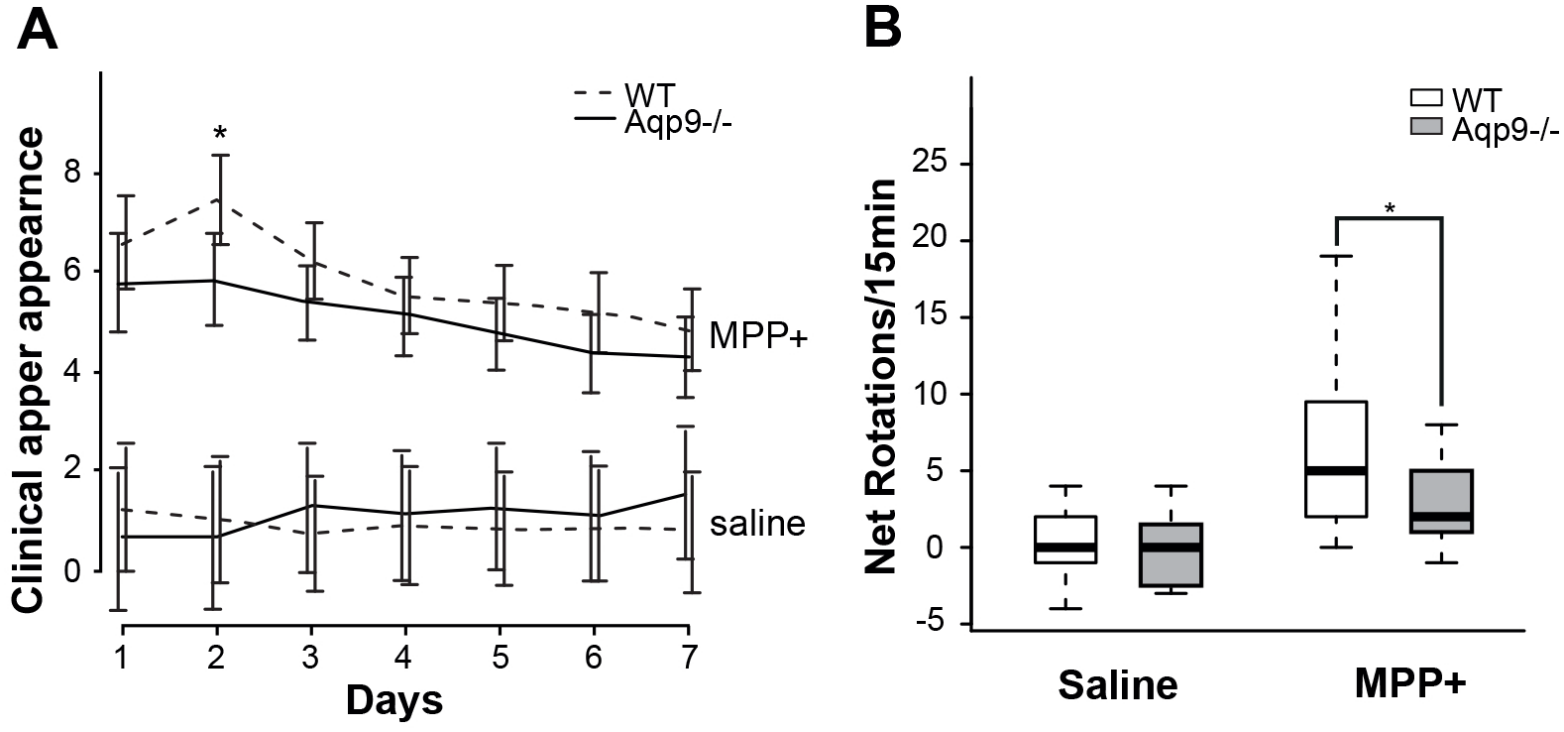
Clinical appearance and behavioral tests of WT and *Aqp9*^-/-^ with unilateral intrastriatal MPP^+^ injection. A) Clinical appearance of animals following intrastriatal injections of MPP^+^ or saline. All animals (MPP^+^: n = 63; saline: n = 21) were assessed daily on a scale from 1–3 for their clinical appearance, including weight loss, inactivity, and reaction patterns, giving a total score ranging from 0–12 (see Results). Following unilateral striatal treatment with MPP^+^, both *Aqp9*^-/-^ and WTs showed a significantly higher score than control animals injected with saline. *Aqp9*^-/-^ mice treated with MPP^+^ showed a lower score than the WT littermates throughout the observation period. Statistical analysis of data from individual observations revealed significant difference on day 2. B) Following a systemic injection apomorphine, *Aqp9*^-/-^ and WT littermates treated with a unilateral striatal injection of 7.5 μg MPP^+^ tend to spin towards the injected side. *Aqp9*^-/-^ (n = 26) show significantly less net rotations (ipsilateral—contralateral turns) compared to WTs (n = 27), with a net rotation number of 2.69 ± 1.91 compared to 6.33 ± 2.15 (mean ± 2 SEM; p = 0.014, n = 53). The rotational behavior was not present in animals treated with saline (n = 17).; Boxes are mean ± 95% CI, bars are maximum and minimum values, *p<0.05.

All animals (MPP^+^: n = 63; saline: n = 21) were evaluated daily and given a score based on post-operative clinical condition, where increasing scores reflected progressive symptoms of disease.. Following unilateral striatal treatment with MPP^+^, both *Aqp9*^-/-^ and WT mice showed a higher score than control animals injected with saline (Fig 6A). In total, 10 MPP^+^ treated animals (6 WT and 4 *Aqp9*^-/-^) were euthanized (n = 4) or died during the three days due to the toxin (n = 6) and were not included in the analysis.

Animals were tested for ipsilateral rotation behavior day six post-surgery after systemic treatment with the dopamine agonist apomorphine. The frequency of rotations is expected to correlate with the extent of dopaminergic cell loss [41]. Significant differences were observed in net rotations (ipsilateral minus contralateral turns) between animals treated with MPP^+^ (n = 53) and saline controls (n = 17). The significance levels were p<0.001 for WT and p = 0.007 for *Aqp9*^-/-^. In the MPP^+^ treated group, net rotational behavior was significantly lower for *Aqp9*^-/-^ animals than for WT littermates (2.69 ± 1.91 and 6.33 ± 2.15, respectively (p = 0.014)) (Fig 6B).

#### HPLC of dopamine and dopamine metabolites

**Fig 7:**
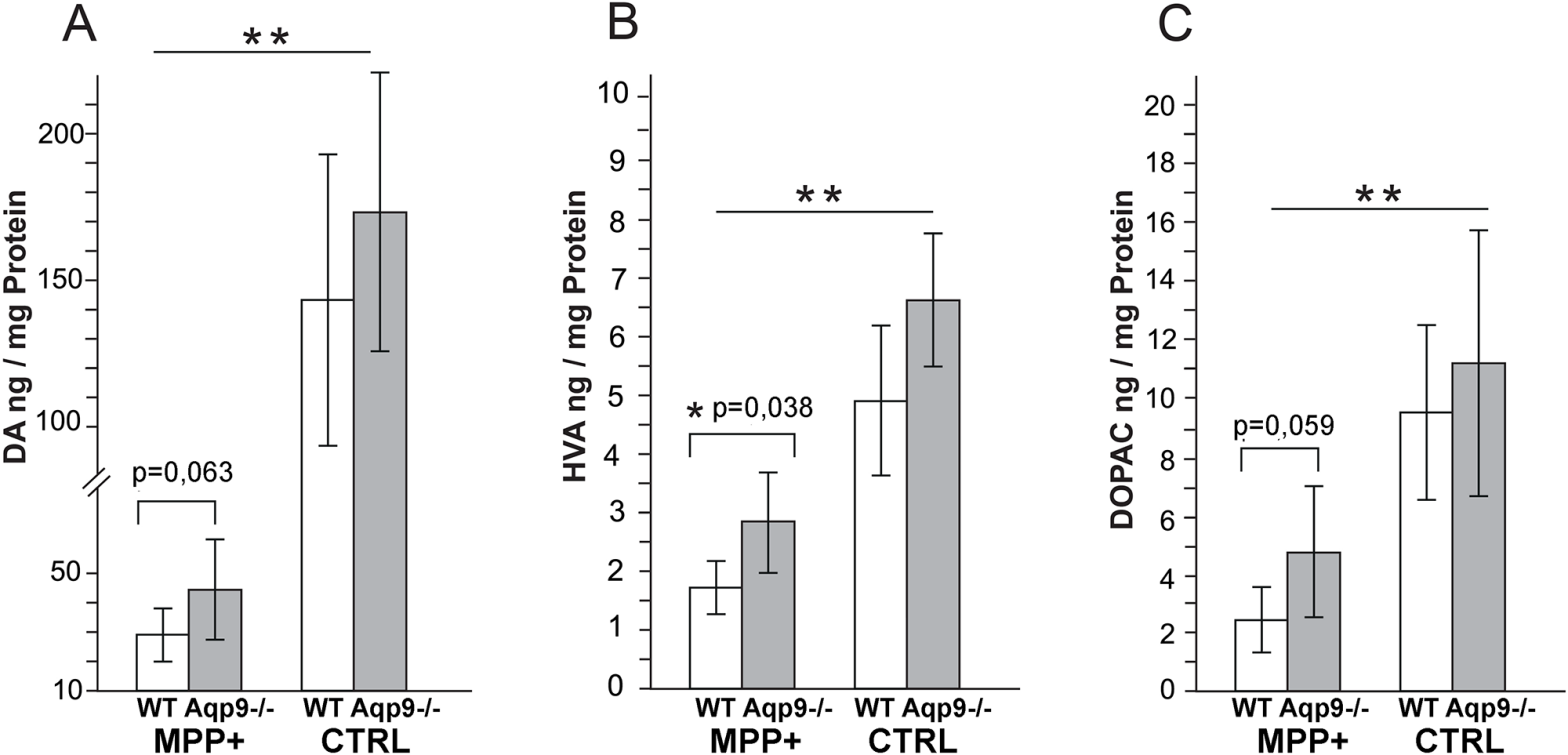
HPLC analysis of dopamine and its metabolites. A-C) After unilateral injections of MPP^+^ in the striatum, the ipsilateral striatum shows a significant reduction in the concentration of DA (A), HVA (B) and DOPAC (C) compared to the contralateral hemisphere (*Aqp9*^-/-^, n = 6; WT, n = 6). The ipsilateral reduction in HVA is significantly more pronounced in WT mice than in *Aqp9*^-/-^ mice (p = 0.038). Corresponding p-values for DA and DOPAC are 0.063 and 0.059, respectively. Bars are mean ± 2 SEM; *p<0.05, ** p<0.01, ***p<0.001.

The levels of dopamine and its metabolites HVA and DOPAC were quantified by HPLC in tissue samples from the ipsi- and contralateral striatum in *Aqp9*^-/-^ (n = 6) and WT littermates (n = 6), seven days after intrastriatal injections of MPP^+^ (Fig 7). MPP^+^ significantly reduced the mean concentration of dopamine (ng/mg protein) on the ipsilateral hemisphere, compared to the contralateral side, by ~83% in WT (p = 0.004) and ~74% in *Aqp9*^-/-^ mice (p = 0.005) (Fig 7A). In WT mice, the reductions in HVA and DOPAC were ~68% (p = 0.003) and ~79% (p = 0.009), respectively. In *Aqp9*^-/-^ mice, metabolite levels were reduced to ~57% (p = 0.002) for HVA and ~57% (p = 0.009) for DOPAC (Fig 7B and 7C). The ipsilateral reduction in HVA was significantly more pronounced in WT mice than in *Aqp9*^*-/-*^ mice (p = 0.038) (Fig 7B). The same tendency was observed for dopamine and DOPAC although the difference was not significant (p = 0.063 and 0.059, respectively) (Fig 7A and 7C).

### Gene expression levels in *Aqp9*^-/-^ animals

**Fig 8:**
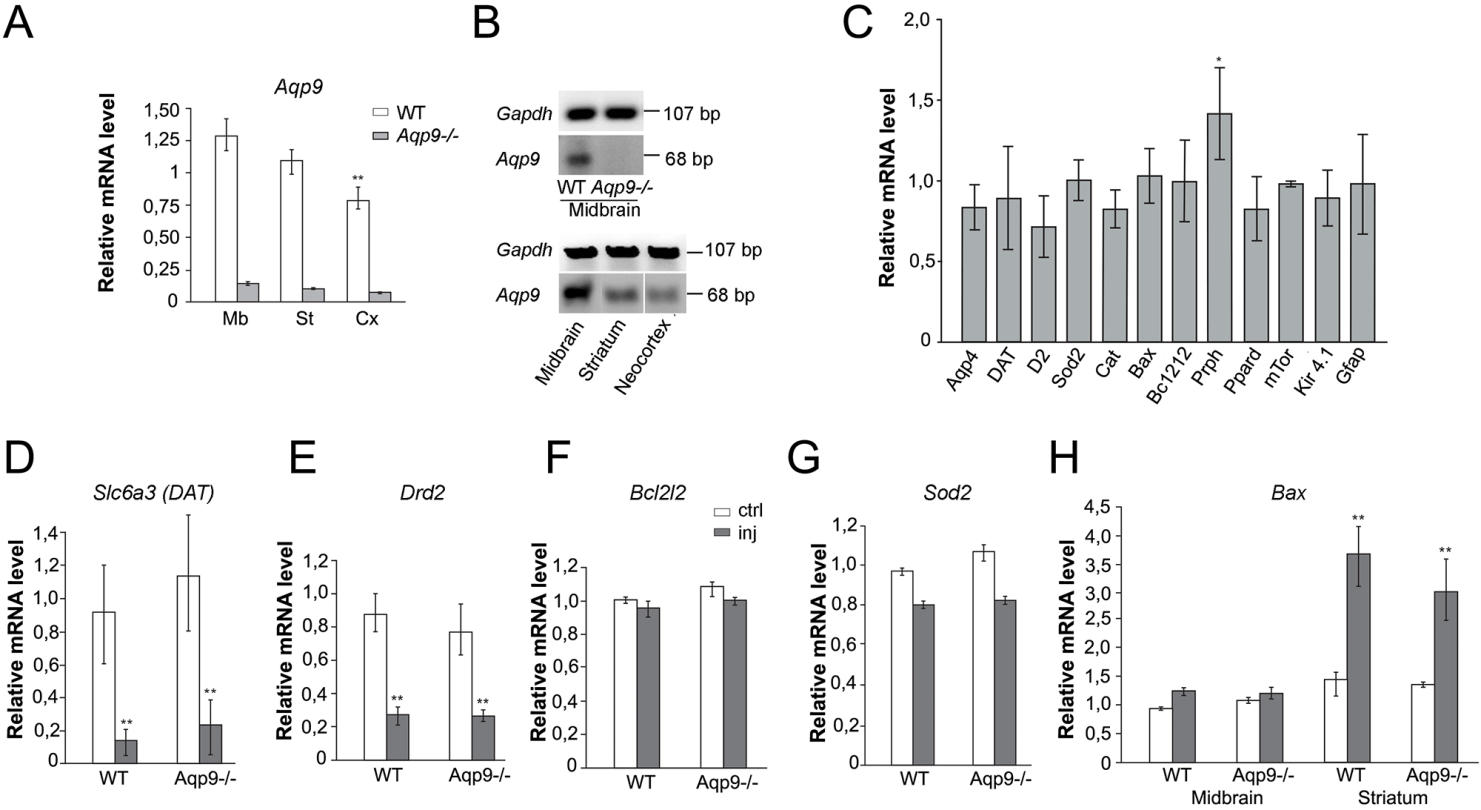
Semiquantitative PCR analyses of gene expression in *Aqp9*^-/-^ and WT mice brain. A) Semi-quantitative Real-Time PCR revealed significantly higher *Aqp9* mRNA levels in the midbrain and striatum than in the neocortex. The level of *Aqp9* mRNA in neocortex is 59% of that in midbrain (p = 0.002). The level of *Aqp9* mRNA in the *Aqp9*^-/-^ mice was close to the detection limit (n = 7 for each genotype). B) Representative DNA agarose gel electrophoresis of midbrain samples from WT and *Aqp9*^-/-^ mice (upper panel), and of three different regions in WT mice (lower panel). These data support the PCR analysis in A. C) In order to rule out that the reduced dopaminergic cell loss in the *Aqp9*^-/-^ mice could be attributed to compensatory up- or downregulation of other genes, an analysis was done of the expression levels of *Aqp4*, *Gfap*, *Kir4*.*1*, *mTOR*, *Prph*, *Cat*, *Ppard*, *Slc6a3 (DAT)*, *Drd2*, *Bcl2*, *Bax* and *Sod2*. The relative levels of these transcripts did not differ between *Aqp9*^-/-^ (n = 7) and WT animals (n = 7). D-H). For selected genes the expression levels were analyzed in the treated and untreated hemispheres. In both groups of animal, the transcript levels of *Drd2* were lower in the striatum on the injected side than in the striatum on the contralateral side (E). In contrast, in both groups of animals, the level of *Bax* was higher in the ipsilateral striatum than in the contralateral one (H). The values indicated in the graphs for *DAT*, *Bcl2 and Sod2* show the values for midbrain, and the values for *Drd2* are for striatum. All values are relative to the values for the corresponding samples from the control hemisphere of the saline treated mice. Bars are mean ± 2 SEM; **p<0.005.

In the knockout animals, the *Aqp9* mRNA level was close to the detection limit, as expected. In WT animals, semi-quantitative real time PCR revealed significantly higher *Aqp9* mRNA levels in the midbrain and striatum (79% of the level in midbrain, p = 0.053) than in the neocortex (59% of the level in midbrain, p = 0.002) (Fig 8A and 8B).

To resolve whether the reduced dopaminergic cell count in the *Aqp9*^-/-^ mice could be attributed to compensatory up- or downregulation of other genes, we analyzed the expression of 12 genes (*Aqp4*, *Gfap*, *Kir4*.*1*, *mTOR*, *Prph*, *Cat*, *Ppard*, *Slc6a3 (DAT)*, *Drd2*, *Bcl2*, *Bax* and *Sod2;* Fig 6C). Only one of these genes (peripherin, *Prph*) showed an increase in expression level (~30%) after *Aqp9* deletion. For five genes (*Slc6a3 (DAT)*, *Drd2*, *Bcl2*, *Bax* and *Sod2)*, mRNA levels were assessed in the ipsi- and contralateral midbrain and striatum of animals injected with MPP^+^. The relative levels of these transcripts did not differ between *Aqp9*^-/-^ and WT animals. In both animals groups, transcript levels of *DAT* and *Drd2* were lower on the injected side than on the contralateral side (Fig 8D–8E). In contrast, in both groups of animals, the level of *Bax* was higher in the ipsilateral striatum than in the striatum contralateral to the injection (Fig 8H).

## Discussion

There is an urgent need to identify the mechanisms underlying the selective vulnerability of dopaminergic cells, the root cause of Parkinson’s disease. In the majority of cases, Parkinson’s disease has no clear genetic etiology, thus emphasizing the importance of unravelling the contribution of environmental factors. Here we show that the parkinsonogenic toxin MPP^+^ permeates AQP9, an aquaglyceroporin water channel that is selectively expressed in dopaminergic neurons, and that a targeted deletion of this water channel affords protection *in vitro* and *in vivo*. We also show that stable expression of AQP9 in HEK cells exacerbates their vulnerability to MPP^+^ and arsenite—another toxin associated with the development of PD. Our data open for the possibility that toxins and other parkinsonogenic substances access dopaminergic neurons through AQP9 and imply that this aquaglyceroporin should be explored as a new target for pharmacological intervention.

The dopamine transporter DAT has long been known to provide an entry route for environmental and experimental toxins [22]. For obvious reasons, this transporter is not relevant as a therapeutic target. AQP9, on the other hand, is not critical to brain function [37] and can be targeted by extant drugs. AQP9 is the first membrane channel that has been associated with the entry of parkinsonogenic toxins.

Our experiments in frog oocytes established that AQP9 is permeable to MPP^+^. To compare the relative significance of AQP9 and DAT we had to resort to cells that normally do not express these molecules, and that by inference are non-dopaminergic. We chose to use HEK cells that have been applied extensively for this purpose in previous studies. We found that AQP9 exacerbates MPP^+^ toxicity, as does DAT. By contrast, the toxicity of arsenite—another parkinsonogenic compound—was potentiated by AQP9 only. The expression of DAT failed to potentiate arsenite toxicity, except at arsenite concentrations exceeding those normally used.

The most salient observation in the present study is that deletion of *Aqp9* affords protection against MPP^+^. We demonstrate a protective effect in organotypic midbrain cultures, as well as after MPP^+^ injections *in vivo*. Stereological quantification of the density of TH-positive neurons in the SNpc after MPP^+^ injection *in vivo* showed a loss of TH-positive neurons of 67% in WT animals, compared with 48% in *Aqp9*^-/-^ animals. The reduced cell loss was accompanied by less pronounced motor dysfunction. The level of the dopamine metabolite HVA in the ipsilateral striatum was about 40% higher in *Aqp9*^-/-^ animals than in WT, supporting the idea that deletion of *Aqp9* helps protect the nigrostriatal pathway against the deleterious effect of MPP^+^.

In organotypic cultures we showed that the protective effect of *Aqp9* gene deletion was mimicked by the application of phloretin—an effective blocker of AQP9. This finding indicates that the reduced vulnerability to MPP^+^ in *Aqp9*^*-/-*^ vs. WT mice is due to the deletion of the *Aqp9* gene and not a result of any off-target effect. In agreement, targeted deletion of *Aqp9* did not alter the expression level of select genes associated with MPP^+^-induced cell death.

Taken together with our data showing increased permeability to MPP^+^ in AQP9-expressing oocytes, these results suggest that AQP9 is involved in mediating the toxicity of MPP^+^ by enabling this toxin to enter dopaminergic neurons. Importantly, as AQP9 is found in the inner mitochondrial membrane as well as in the plasma membrane [23], this channel may allow MPP^+^ and other toxins to permeate the mitochondrial matrix. This is relevant, as the electron transport chain has been identified as one of several targets of parkinsonogenic toxins [47,48].

### Methodological considerations

Here we used an *in vivo* model of Parkinson’s disease where MPP^+^ was injected unilaterally into the striatum of *Aqp9* deficient and WT mice. The injections covered a large part of the projection field of nigral dopaminergic neurons and thus allowed retrograde transport of toxin to these neurons. In designing these experiments we aimed for a cell loss of more than 60% in the SNpc of WT mice, comparable to the cell loss seen in postmortem analyses of patients with Parkinson’s disease [49]. The dose of MPP^+^ produced a rather extensive and unspecific damage around the injection site. In midbrain, however, the deleterious effect of MPP^+^ was largely restricted to the SN. Specifically, neither genotype showed a substantial loss of dopaminergic cells in the VTA. The most likely explanation is that the MPP^+^ injection was discrete enough to encompass the projection area of nigral neurons without encroaching upon the projections of those dopaminergic neurons that reside in the VTA.

Compared with WT, *Aqp9*^-/-^ mice developed less motor dysfunction when exposed to MPP^+^. In the apomorphine rotation test, *Aqp9*^-/-^ mice showed significantly less ipsilateral spinning than WT littermates. Apomorphine is thought to induce contralateral spinning at a high rate due to supersensitivity of dopaminergic D_2_ receptors in the striatal nerve terminals [41]. However, several studies have shown that severe damage to the striatum—similar to that seen here—induces ipsilateral turning behavior following administration of apomorphine [50–54]. In agreement, we observed a pronounced decrease in the expression level of dopamine D_2_ receptors in the striatum of both genotypes, reflecting the extensive damage and explaining the ipsilateral rotation.

In line with the outcome of the apomorphine rotation test, the clinical appearance of MPP^+^ injected animals, as well as the pronounced drop in dopamine and dopamine metabolites in the ipsilateral striatum, point to a rather severe local effect of MPP^+^ injections. Histological analyses concurred by showing areas of cell loss and immune cell infiltration involving large parts of the striatum and overlying cortex. The lesion did not extend into the ventral striatum. The nonspecific nature of the local lesion was distinct from the lesion in midbrain, which likely is mediated by retrograde transport of the injected toxin. Thus, the midbrain lesion specifically involved TH-positive neurons in the SNpc, leaving nearby VTA neurons (that normally project to the ventral striatum) virtually unharmed.

AQP9 is expressed in dopaminergic midbrain neurons [23,24,26,55] but is also found in astroglia throughout the brain [23,25,55,56]. This explains why we found *Aqp9* mRNA in other brain regions, including the neocortex. However, our regional analysis showed higher levels of *Aqp9* mRNA in midbrain and striatum than in neocortex. This is consistent with previous immunocytochemical data and with the finding that these structures contain a neuronal as well as an astroglial pool of AQP9 [26].

Acute injections of MPP^+^ remain a rather crude model for Parkinson’s disease. This calls for due care when it comes to the interpretation of the present results. In a clinical setting Parkinson’s disease develops over years, even decades, implying that the present study should be followed up in more chronic models. However, even with long time exposure to minute amounts of a putative neurotoxin the vulnerability may be determined by the same factors as in the acute model *i*.*e*., the transport capacity for the toxin or toxins in question. Thus, the expression level of AQP9 in plasma and mitochondrial membranes might not only explain the selective vulnerability of dopaminergic neurons within an individual, but possibly also the differences in vulnerability between individuals. This issue will be pursued in future studies.

### AQP9 vs. DAT

In earlier works the selective sensitivity of SN neurons to parkinsonogenic toxins has been attributed to the activity of the dopamine transporter DAT [22]. It is well documented that DAT transports MPP^+^ [57]. This begs the question whether DAT is the predominant mediator of MPP^+^ toxicity, leaving only a minor role for AQP9. We addressed this issue by use of the MTT assay, which is widely employed to assess the effects of toxins, including MPP^+^.

We performed the MTT assay in HEK293 cells as these cells do not normally express AQP9 or DAT. HEK293 cells stably transfected with *h*AQP9 were more sensitive to MPP^+^ than were cells stably transfected with *h*DAT. Notably, while 1 μM MPP^+^ was required to produce significant effects in *h*DAT expressing cells, 0.1 μM MPP^+^ was sufficient to induce toxicity in cells expressing *h*AQP9. HEK293 cells expressing *h*AQP9 were also clearly more sensitive to the parkinsonogenic compound arsenite than were HEK293 cells expressing *h*DAT. At concentrations up to 10 μM, arsenite had no toxic effects in *h*DAT expressing HEK293 cells. This is in line with previous studies that did not detect any difference in arsenite sensitivity between *h*DAT expressing HEK293 cells and native HEK293 cells [58]. Only at very high arsenite concentrations do *h*DAT expressing cells differ from native HEK293 cells in terms of their viability.

In our *in vivo* model, MPP^+^ caused a significant dopaminergic cell loss also in the *Aqp9*^-/-^ animals. This can probably be attributed to the expression of DAT in the nigrostriatal terminals, facilitating uptake of MPP^+^ also into dopaminergic neurons that are depleted of AQP9 [22]. In midbrain organotypical cultures, the nigrostriatal axons are severed, implying that MPP^+^ uptake through AQP9, expressed at high levels in dopaminergic cell bodies [23,26], may predominate over MPP^+^ uptake by DAT. This may explain why the protective effect of *Aqp9* deletion was more pronounced in the slice cultures than in the *in vivo* model.

We conclude that both DAT (a transporter) and AQP9 (a channel) are likely to be involved in mediating MPP^+^ toxicity. However, while DAT transports MPP^+^, it has a rather narrow substrate specificity that does not encompass the wide range of toxins that have been associated with the development of Parkinson’s disease. Unlike DAT, which is essential for normal brain function, AQP9 is not critically involved in any known physiological process in brain. Thus, *Aqp9*^-/-^ mice have a rather mild phenotype largely limited to the peripheral organs such as the liver [37]. Little is known about the roles of AQP9 in human brain as most studies of AQP9 in humans have focused on the reproductive system and placenta [59–61] If applicable to humans, the present data suggest that pharmacologic inhibition of AQP9 could be considered a viable approach to curb the progression of Parkinson’s disease in cases where exposure to exogenous toxins is documented or suspected as a cause of morbidity.

Importantly, targeted deletion of *Aqp9* did not affect the expression level of DAT. Our finding that phloretin treatment mimicked the effect of *Aqp9* deletion lends further support to the idea that the protection observed is a bona fide effect of the targeted deletion and not due to compensatory up- or downregulation of other genes.

## Conclusions and prospects for new therapy

Earlier studies have shown that AQP9 is expressed in midbrain neurons that contain TH—a marker of dopaminergic neurons [23,24,26,55]. Here, using both *in vitro* and *in vivo* models, we provide evidence that AQP9 contributes to the selective vulnerability of dopaminergic neurons to exogenous toxins. This is the first study to couple this vulnerability to a specific membrane channel allowing toxin influx. The broad substrate specificity of AQP9 implies that that this channel might permit entry of a number of compounds that compromise the viability of dopaminergic neurons. It is of interest in this regard that the *Aqp9* gene contains a negative insulin response element, and that insulin downregulates its expression level [62–64]. Studies on experimental diabetes in rodents as well as tissue samples from patients with diabetes have shown an increase in the level of AQP9 mRNA and protein [65,66]. The possibility should be considered that an upregulation of *Aqp9* contributes to the increased prevalence of Parkinson’s disease among patients with diabetes mellitus type 2 [67].

As pointed out in our discussion, the rather short survival times that are used in such models do not match the protracted course of disease development in the human condition. This is a general shortcoming of most current animal models of PD and calls for due care when it comes to the interpretation of the results. Inspired by the comments of the reviewers we deepen our discussion of this point in our revised text. As we state in our discussion a natural follow up of our study is to subject our *Aqp9-/-* animals to other and more chronic experimental approaches that more faithfully mimic the progressive and protracted nature of human disease.

## Supporting information

S1 Fig Tissue damage and immune cell infiltration following ipsilateral intrastriatal injection of MPP^+^. A-D) Semi-thin sections immunostained for TH (A) or hematoxylin/eosin (B-D) showing local effect of the MPP^+^ injections. The site of MPP^+^ injection (Ipsi) in the anterior striatum is indicated by arrow (A). The toxin induces tissue damage in the entire ipsilateral striatum (CPu). Cell loss and immune cell infiltration extended into parts of the overlying neocortex (B-D). Reflecting the tissue damage, the TH immunosignal was blurred, with loss of the sharp contrast between the corpus callosum (cc), overlying cortex (Cx) and striatum (CPu) seen on the contralateral (Cont) side. Stippled line outlines the damaged area. Boxed areas in B are enlarged in C and D. cc, Corpus Callosum; CPu, Caudate-Putamen; Cx, frontal cortex; LV, Lateral Ventricle. Scale bar: 1000 μm.
(TIF)

S1 File Methods. (DOCX)

S2 File Humane endpoints checklist. (DOCX)

S1 Table List of the TaqMan probes used for quantitative RT-PCR. (DOCX)

S2 Table Overview of the estimated total cell count of TH-positive neurons. (DOCX)

## Abbreviations

AQP4: aquaporin-4
AQP9: aquaglyceroporin-9
DAB: 3,3'-diaminobenzidine
DAT: dopamine transporter
DMSO: dimethyl sulfoxide
DOPAC: 3,4-dihydroxyphenylacetic acid
EGFP: enhanced green fluorescent protein
HEK: human embryonic kidney cells 293
HPLC: High performance liquid chromatography
HVA: homovanillic acid
MPP^+^: 1-methyl-4-phenylpyridinium
MTT: 3-(4,5-dimethylthiazol-2-yl)-2,5-diphenyltetrazolium bromide
PBS: phosphate buffered saline
SN: substantia nigra
SNpc: substantia nigra pars compacta
SNpr: substantia nigra pars reticulata
TH: tyrosine hydroxylase
VTA: ventral tegmental area
WT: wild type
YFP: yellow fluorescent protein
