# Supplementary figures and images for: Targeted deletion of the aquaglyceroporin AQP9 is protective in a mouse model of Parkinson’s disease
Source: PLoS One. 2018 Mar 22;13(3):e0194896. doi: 10.1371/journal.pone.0194896 (PMC5864064; doi:10.1371/journal.pone.0194896)

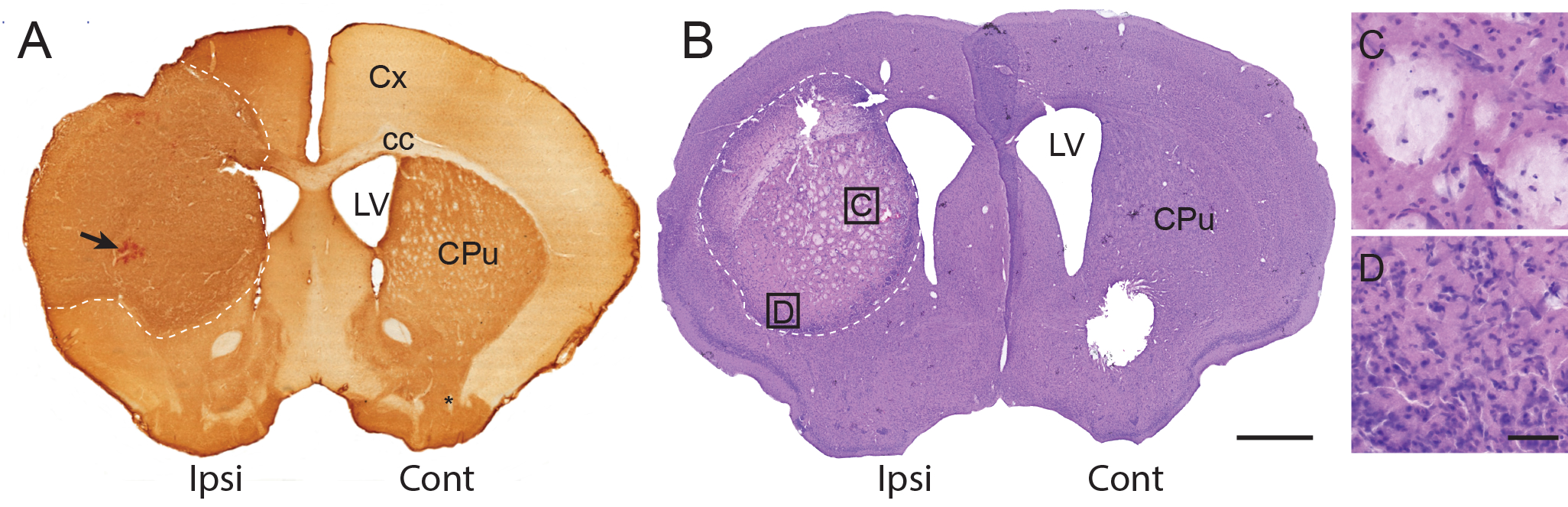

Supplement: S1 Fig — A-D) Semi-thin sections immunostained for TH (A) or hematoxylin/eosin (B-D) showing local effect of the MPP+ injections. The site of MPP+ injection (Ipsi) in the anterior striatum is indicated by arrow (A). The toxin induces tissue damage in the entire ipsilateral striatum (CPu). Cell loss and immune cell infiltration extended into parts of the overlying neocortex (B-D). Reflecting the tissue damage, the TH immunosignal was blurred, with loss of the sharp contrast between the corpus callosum (cc), overlying cortex (Cx) and striatum (CPu) seen on the contralateral (Cont) side. Stippled line outlines the damaged area. Boxed areas in B are enlarged in C and D. cc, Corpus Callosum; CPu, Caudate-Putamen; Cx, frontal cortex; LV, Lateral Ventricle. Scale bar: 1000 μm. (TIF) [file pone.0194896.s001.tif]
