# Supplementary material for: Targeted deletion of the aquaglyceroporin AQP9 is protective in a mouse model of Parkinson’s disease
Source: PLoS One. 2018 Mar 22;13(3):e0194896. doi: 10.1371/journal.pone.0194896 (PMC5864064; doi:10.1371/journal.pone.0194896)
Supplement: S1 File — (DOCX) [file pone.0194896.s002.docx]

# S1 Methods:

## Histological and stereological evaluation of MPP^+^ lesions and TH-positive neurons

Seven days post-surgery, animals (n=27) were deeply anaesthetized (zoletil mixture; 0.1 ml/10 g; intraperitoneally) and perfusion-fixed with 4% formaldehyde. Brains were dissected, post-fixed in 4% formaldehyde overnight, and stored in 0.4% formaldehyde at 4°C. The control hemisphere was marked with a shallow cut in the dorsal midbrain to allow discrimination from the treated hemisphere. Following cryo-protection by incubation in 10%, 20% and 30% sucrose, brains were coronally sectioned at 40 µm using a HM 450 freeze microtome (Microm, Walldorf, Germany). Sections through the striatum and SN were stained with a standard hematoxylin/eosin (H+E) protocol to visualize morphological and pathological features (S1 Fig). In order to visualize TH positive cells, successive sections through the SN were immunostained with mouse anti-TH (1:1000, Chemicon) followed by incubation with Streptavidin-Biotinylated horseradish peroxidase complex (1:100; GE Healthcare). Sections were further incubated with DAB (Sigma Aldrich) dissolved in 0.1 M PBS for 5 minutes, then treated with a DAB-solution containing 0.03% H_2_O_2_ (Applichem, Darmstadt, Germany). Histological images were captured through a 20× objective using a slide scanner (AxioScan, Carl Zeiss Microimaging Gmbh, Jena, Germany).

The location and extent of MPP^+^ injection sites were evaluated in images of H+E and immunostained striatal sections. TH positive neurons were stereologically quantified bilaterally in the SNpc, SNpr, and VTA of *Aqp9*^-/-^ mice and WT controls injected with MPP^+^ (*Aqp9*^-/-^, n=8; WT, n=7) or saline (*Aqp9*^-/-^, n=3; WT, n=3). Sections from the rostral midbrain were stained with 0.1% toluidine blue (Sigma Aldrich) and the rostral end of SNpc (Bregma -2.7 mm) identified by reference to Paxinos and Franklin’s Atlas of the Mouse Brain [1]. For stereological quantification, 10 TH-immunostained sections were systematically sampled at 120 µm intervals covering the entire SN (Bregma -2.70 to -3.80 [1]) and investigated using an Olympus Bx52 microscope (Olympus, Tokyo, Japan) equipped with a motorized stage (LEP MAC5000, LUDL Electronic Products Ltd., Hawthorne, NY, USA), and Optronics MicroFire digital camera (Optronics Goleta CA, USA) running the StereoInvestigator 7 software (MicroBrightField Inc, Williston, VT, USA). Regions of interest for stereological analysis, defined by the presence of TH positive neurons, were drawn in the SNpc, SNpr, and VTA contralateral to the MPP^+^ injections using a 4× objective (Olympus UPlanApo, NA 0.16). TH labeled cells were identified and counted through a 40× objective (Olympus UPlanApo, NA 0.85) using the Optical Fractionator approach [2]. Optical dissector probes (100×100×20 µm) yielding an average of six cells per frame were positioned within the regions of interest on an isotropic grid with gridlines spaced at 150 µm. TH labeled neurons with visible nuclei were counted if their somata were within or touching the inclusion lines, without touching the exclusion lines of the virtual counting frame. For each region of interest, the estimated density of labeled cells was calculated as the total number of cells within each volume based on mean section thickness, together with Gundersen coefficient of error m=1 for assessment of the precision of the estimate [3].

## RNA isolation and real time qPCR

Seven days post-surgery, animals (MPP^+^, n=7 for each genotype; saline, n=3 for each genotype) were decapitated under isofluorane anesthesia and the brains were dissected out. The brains were cut along the midsagittal line into the injected hemisphere and control hemisphere. Midbrain, striatum and neocortex were dissected out from both hemispheres and snap frozen. The samples were stored at -80°C until analysis.

Total RNA was isolated from the regional brain samples using the RNeasy Mini Kit (QIAGEN, Hilden, Germany) including the on column DNase digestion. The RNA concentration and integrity were determined using a NanoDrop 2000c spectrophotometer (Thermo Scientific) and ethidium bromide visualization after agarose gel electrophoresis. For each regional sample, the same amount of RNA was reverse-transcribed with Oligo (dT)_15_ using the GoScript Reverse Transcription System (Promega, Madison, USA) according to the manufacturer's instructions. All the cDNA samples were diluted in 10 mM Tris-HCl (pH 8.0) to 5 ng/μl. Real-Time PCR was carried out in a total volume of 20 µl, containing 2 x Universal Master Mix II without UNG (Applied Biosystems, Foster City, CA, USA), 20 x corresponding TaqMan gene expression assay FAM-MGB conjugated (Applied Biosystems) and 2 µl cDNA samples. Thermal cycling was performed on the StepOnePlus system (Applied Biosystems) with the following conditions: 95°C for 10 minutes, followed by 40 cycles of 95°C for 15 seconds and 60°C for 1 minute. The experiments were carried out in duplicate for each data point. Using the NormFinder software, *Gapdh* was used as an internal control for normalization of the gene expression. List of the TaqMan probes used in the study are shown in S1 Table.

The relative change in gene expression (ΔΔCt) was used for comparison of the gene expression in the MPP^+^-treated hemisphere versus control hemisphere. Data were analyzed using DataAssist Software v3.01 (Applied Biosystems). For each analysis, corresponding samples from the control hemisphere of the saline injected WT animals were used as the calibrator. To visualize the expression of *Aqp9* in WT and *Aqp9^-/-^* animals, we used End-Point PCR. The reaction included the GoTaq Green (Promega) master mix, *Aqp9* probe and 50 ng cDNA template in the total volume of 20 µl. *Gapdh* was used as control using the same cDNA diluted to 2.5 ng/ul. The PCR was done at 95°C (10 minutes) followed by 30 cycles at 95°C (30 seconds), 55°C (30 seconds) and 72°C (2 minutes). The products were visualized on an ethidium bromide agarose gel.

## Supporting references

1. Paxinos GaF, K (2007) The Mouse Brain in Sterotaxic Coordinates: Elsevier Inc.

2. Gundersen HJ, Bagger P, Bendtsen TF, Evans SM, Korbo L, et al. (1988) The new stereological tools: disector, fractionator, nucleator and point sampled intercepts and their use in pathological research and diagnosis. APMIS 96: 857-881.

3. Gundersen HJ, Jensen EB, Kieu K, Nielsen J (1999) The efficiency of systematic sampling in stereology--reconsidered. J Microsc 193: 199-211.
