# Supplementary material for: Targeted deletion of the aquaglyceroporin AQP9 is protective in a mouse model of Parkinson’s disease
Source: PLoS One. 2018 Mar 22;13(3):e0194896. doi: 10.1371/journal.pone.0194896 (PMC5864064; doi:10.1371/journal.pone.0194896)
Supplement: S1 Table — (DOCX) [file pone.0194896.s004.docx]

| **Assay ID** | **Gene name** | **Exon boundary** |
| --- | --- | --- |
| Mm00508094_m1 | *Aqp9* | 1-2 |
| Mm00438388_m1 | *Slc6a3* | 3-4 |
| Mm00438545_m1 | *Drd2* | 7-8 |
| Mm01313000_m1 | *Sod2* | 4-5 |
| Mm00432054_m1 | *Bcl2l2* | 3-4 |
| Mm00432051_m1 | *Bax* | 5-6 |
| Mm00802131_m1 | *Aqp4* | 4-5 |
| Mm00437992_m1 | *Cat* | 8-9 |
| Mm00449699_g1 | *Prph* | 2-3 |
| Mm00803184_m1 | *Ppard* | 5-6 |
| Mm00444968_m1 | *Mtor* | 6-7 |
| Mm00445028_m1 | *Kcnj10* | 1-2 |
| Mm01253033_m1 | *Gfap* | 6-7 |
| Mm99999915_g1 | *Gapdh* | 2-3 |
