# Supplementary material for: Targeted deletion of the aquaglyceroporin AQP9 is protective in a mouse model of Parkinson’s disease
Source: PLoS One. 2018 Mar 22;13(3):e0194896. doi: 10.1371/journal.pone.0194896 (PMC5864064; doi:10.1371/journal.pone.0194896)
Supplement: S2 Table — (DOCX) [file pone.0194896.s005.docx]

| **Region** | **Treatment** | **Genotype** | **Hemisphere** | **TH+ cell Count** | **SEM** |
| --- | --- | --- | --- | --- | --- |
| SNpc | MPP^+^ | KO | ctrl | 2881.43 | 178.75 |
|  |  |  | inj | 1504.55 | 114.30 |
|  |  | WT | ctrl | 3291.83 | 172.03 |
|  |  |  | inj | 1071.72 | 111.85 |
|  | saline | KO | ctrl | 4649.28 | 317.56 |
|  |  |  | inj | 4543.77 | 150.52 |
|  |  | WT | ctrl | 4161.63 | 265.04 |
|  |  |  | inj | 4128.72 | 122.76 |
| VTA | MPP^+^ | KO | ctrl | 2127.89 | 146.67 |
|  |  |  | inj | 2000.10 | 117.93 |
|  |  | WT | ctrl | 2174.06 | 117.47 |
|  |  |  | inj | 1743.72 | 134.79 |
|  | saline | KO | ctrl | 3022.41 | 188.16 |
|  |  |  | inj | 2876.37 | 264.01 |
|  |  | WT | ctrl | 2137.18 | 43.23 |
|  |  |  | inj | 2149.65 | 94.44 |
